# Developing and implementing a novel mentorship model (4^+ 1^) for maternal, newborn and child health in Rwanda

**DOI:** 10.1186/s12913-020-05789-z

**Published:** 2020-10-07

**Authors:** Anaclet Ngabonzima, Cynthia Kenyon, Celestin Hategeka, Aimee Josephine Utuza, Paulin Ruhato Banguti, Isaac Luginaah, David F Cechetto

**Affiliations:** 1 Economic Community of Central African States (ECCAS), Libreville, Gabon; 2 Department of Anatomy & Cell Biology, Schulich School of Medicine & Dentistry, University of Western Ontario, London, Ontario N6A 5C1 Canada; 3 Neonatal - Perinatal Medicine, University of Western Ontario, 800 Commissioners Rd E, D4-200, London, Ontario N6A 5W9 Canada; 4 Centre for Health Services and Policy Research, School of Population and Public Health, Faculty of Medicine, Department of Global Health and Population, Harvard TH Chan School of Public Health, Boston, MA USA; 5 University of Western Ontario, London, Ontario N6A 5C1 Canada; 6 College of Medicine and Health Sciences (CMHS), University of Rwanda, Kigali, Rwanda; 7 Department of Geography, University of Western Ontario, London, Ontario N6A 5C1 Canada

**Keywords:** Mentorship, Maternal, Neonatal, Rwanda, Interprofessional collaboration

## Abstract

**Background:**

There are a number of factors that may contribute to high mortality and morbidity of women and newborns in low-income countries. These include a shortage of competent health care providers (HCP) and a lack of sufficient continuous professional development (CPD) opportunities. Strengthening the skills and building the capacity of HCP involved in the provision of maternal, newborn and child health (MNCH) is essential to ensure quality care for mothers, newborns and children. To address this challenge in Rwanda, mentorship of HCPs was identified as an approach that could help build capacity, improve the provision of care and accelerate the reduction in maternal and neonatal mortality and morbidity.

In this paper, we describe the development and implementation of a novel mentorship model named Four plus One (4^+ 1^) for MNCH in Rwanda.

**Methods:**

The mentorship model built on the basis of inter-professional collaboration (IPC) was developed in early 2017 through consultations with different key actors. The design phase included refresher courses in specific skills and training course on mentoring. Field visits were conducted in 10 hospitals from June 2017 to February 2020. Hospital management teams (MT) were involved in the development and implementation of this mentorship model to ensure ownership of the program.

**Results:**

Upon completion of planned visits to each hospital, a total of 218 HCPs were involved in the process. Reports prepared by mentors upon each mentorship visit and compiled by Training Support and Access Model (TSAM) for MNCH’CPD team, highlighted the mothers and newborns who were saved by both mentors and mentees. Also, different logbooks of mentees showed how the capacity of staff was strengthened, thereby suggesting effectiveness of the model. Through different mentorship coordination meetings, the model was much appreciated by the MTs of hospitals, especially the IPC component of the model and confirmed the program ‘effectiveness.

**Conclusion:**

The initiation of a mentorship model built on IPC together with the involvement of the leadership of the hospital may be the cause effect of reduction of specific mortality and improve MNCH in low resource settings even when there are a limited number of specialists in the health facilities.

## Background

Perinatal mortality remains unacceptably high in low income countries (LICs). According to 2015 World Health Organization (WHO) data; 303,000 women died in 2015 from pregnancy or childbirth-related complications, representing 830 maternal deaths per day. In addition, 2.5 million newborns died in 2017 and a very high number of infants were stillborn, approximately 18.4 stillbirths for every 1000 births [1]. Almost all these deaths occurred in low resource settings and a significant proportion of them could have been prevented with timely access to quality care [2, 3]. The leading causes for maternal deaths include post-partum hemorrhage, preeclampsia and eclampsia, and infection, while perinatal asphyxia, prematurity and neonatal infections are the leading causes of neonatal mortality [4].

In LICs, lack of competent HCPs to provide improved perinatal care is one of contributing factors to most maternal and newborn deaths. This shortage of primary HCPs is compounded by lack of specialists such as anesthesiologists, pediatricians and obstetricians, as well as a maldistribution of staff, inadequate training and lack of ongoing educational opportunities, skill mix imbalance, recruitment and retention challenges, increasingly complicated ‘first world’ medical problems and the challenging social-cultural-political-economic context [5–9].

Although, the lack of health care professionals and related problems are major concerns, there are also gaps in implementation of effective continuing professional development (CPD) to build competence and confidence in clinical care. Gaps also exist in management and leadership training, team building, professionalism, interpersonal communication, teaching, and accountability. These gaps suggest the need to identify and invest in effective models to better train and support health workers [10, 11].

For decades, HCPs in LICs have been trained on interventions supported by NGOs with a focus on specific health problems. These training sessions have mostly been done off-site, often in hotels far away from the participant’s place of work. Generally, only one or two health care providers from an institution tend to be trained, thus, making it challenging for the newly trained HCPs to institute significant change. Resources have not usually been available to provide ongoing support for HCPs who receive new training [12]. Consequently, it has been difficult to demonstrate that these kinds of remote interventions produce lasting change in practice or improvement in health outcomes. In addition, such modular training programs do not address system level barriers including poor service organization, lack of team work, lack of communication among health care providers, and lack of recognition of required essential urgent drugs. Also, these modules tend not be based on the needs and gaps of health care providers [5, 6, 9, 10].

Existing evidence highlights that didactic training alone is not effective in transferring new knowledge into practice sustainably. For example, Emergency Obstetrical and Neonatal Care (EmONC) training was demonstrated to be effective in improving the knowledge and skills of HCPs. But without ongoing support and follow-up, the evidence showed the effectiveness of single training is limited. Thus, although didactic training improves the clinical knowledge of the participants, translating this into improved outcomes in practice is difficult or impossible to demonstrate [5].

Although individual skill training is a critical, interprofessional collaboration (IPC) is also essential. Maternal care is complex and unexpected emergencies frequently occur. Success in managing emergency situations requires teamwork and good communication [5]. Research in the UK has identified that lack of teamwork is an important factor in suboptimal delivery of care. Maternity staff may feel they have a high level of competence but when faced with an urgent situation, they may realize that are not skilled in teamwork or may find the team they are working with is unfamiliar to them. Traditionally HCPs do not train together, usually doctors and nurses or midwives attend separate sessions. Therefore, HCPs from different professional groups may have been trained using different algorithms or procedures making team functioning during an emergency challenging [13].

Research has also demonstrated the impact of IPC in strengthening the quality of care, establishing a climate of good communication, improving patient safety, and in reducing the culture of blame leading to overall improved patient care. A 2018 Cochrane review identified teamwork, communication, collaboration, and building a climate of trust as factors contributing to the quality of care provided by birth attendants [13, 14].

In high-income countries mentorship is considered essential to support the professional development of health professions faculty at all levels, and to role model professionalism for students in the health professions. Although research in LICs is limited, mentorship programs have been shown to improve quality of care [15–17]. Mentorship has been identified as a teaching and learning process that can assist HCPs working in a complex clinical setting to provide quality health care services [6, 9, 10, 16–20]. A mentorship program could support HCPs in rural areas in ways that the traditional hierarchical supervision model does not. On-site mentorship may provide support to HCPs in their workplace and help accelerate mortality and morbidity reduction through capacity building [21]. Making use of interprofessional teams of mentors could also greatly enhance the overall communication and impact of the mentoring experience.

We describe below development and implementation of a mentorship framework or model called four plus one (4^+ 1^) that can be used in settings in which the supply of HCPs is challenging and interprofessional collaboration is essential.

This paper aims to describe the development, implementation as well as the results of the novel mentorship model (4^+ 1^) for health care providers providing maternal and neonatal care in 10 hospitals located in 6 districts of Rwanda.

### Intervention context

In Rwanda, high impact interventions in combination with political support and enhanced technical abilities, have allowed the country to make great strides in the area of women’s and children’s health. Rwanda was one nine countries to reach Millennium Development Goals (MDGs) related to the reduction of maternal deaths. Other countries include Bhutan, Cabo Verde, Cambodia, Iran, Lao People’ Democratic Republic, Maldives, Mongolia and Timor-Leste. In Rwanda, the Maternal Mortality Ratio (MMR) declined from 1071 per 100,000 live births in 2000 to 210 per 100,000 live births in 2015, thus achieving MDG 5. The country also achieved the MDG 4 related to under 5 mortality. The under-five mortality declined from 196 per 1000 live births to 50 during the same time frame [22].

Even though significant improvements have been made in maternal, newborn and child health (MNCH), the number of maternal deaths remains high. Facility-based audits have shown that 70% of maternal deaths in Rwanda are due to direct causes, with postpartum haemorrhage the leading direct cause (23%) followed closely by postnatal infection and eclampsia [22]. In addition, neonatal mortality is high and the reduction in newborn deaths has not followed the same downward trend seen in other age groups in under-five children [23]. The main causes of neonatal death in Rwanda are preterm birth complications, intrapartum-related complications leading to birth asphyxia, and neonatal infections [22]. Most deaths occur either directly associated with complications of labour or on the day of birth from other causes. An estimated 73% of all neonatal deaths occur in the first week of life [24].

In fact, even though 91% of all deliveries occur in health facilities (health centers and hospitals), large proportion of maternal and newborn deaths take place in hospitals [22]. In addition, a review of death records revealed that most maternal and newborn deaths tend to be associated with a delay in receiving quality care [24]. Therefore, in most cases, it is not a mother’s ignorance nor their lack of ability to get to a facility that is the main barrier to receiving quality of care but it is the delay within the health facility which is the cause of negative health outcomes; if one considers that a substantial proportion of maternal and newborn deaths takes place in these facilities. Thus, a major area of improvement that would accelerate the reduction of maternal, perinatal and child deaths is by improving the provision of quality health care within facilities. Therefore, mentorship can be used as an intervention to reduce maternal, newborn and child deaths through the provision of quality services during perinatal period by building the capacity of HCPs [25].

In 2016, the Rwanda Ministry of Health and the Rwanda Biomedical Centre adopted the mentorship approach to further strengthen the health care system [26]. The goal of this approach was to provide HCPs with practical skill enhancement through on-site mentorship provided by experienced professionals. Mentors were selected based on their clinical expertise as well as their interpersonal skills and ability to motive others [26].

The program described below was developed for implementation in Rwanda by the Training, Support & Access Model (TSAM) for MNCH project. TSAM for MNCH is a project focused on improving Maternal, Newborn and Child Health (MNCH) in Rwanda and is funded by Global Affairs Canada. TSAM is a collaborative partnership between the University of Western Ontario, the University of Rwanda, the Rwanda Medical and Dental Council, and the National Council of Nurses & Midwives in Rwanda. Other collaborating organizations include the Rwanda Society of Obstetricians & Gynaecologists, the Rwanda Paediatric Association, the Rwanda Association of Midwives and the Rwanda Society of Anesthesiologists. The program builds on the goal of the Rwanda Ministry of Health to improve safe delivery of emergency care in MNCH in Rwanda through mentorship.

## Methods

### Aim

This paper aims to describe the development, implementation as well as the results of the novel mentorship model (4^+ 1^) for health care providers providing maternal and neonatal care in 10 hospitals located in 6 districts of Rwanda.

### Design and settings

The implementation of the model was done in three districts of Northern Province of Rwanda (Rulindo, Gicumbi and Gakenke) and 3 districts of Southern Province of Rwanda (Muhanga, Gisagara and Ruhango) These 6 districts were assigned to the TSAM project as per the Memorandum of Understanding between the project and the Ministry of Health.

The mentorship model was developed in early 2017 through consultations with different key actors and is based on inter-professional collaboration (IPC) with a team of five mentors, including a gynecologist/obstetrician, midwife, pediatrician, pediatric nurse and an anesthesiologist, giving rise to the name four plus one (4 + ^1^) to recognize the inclusion of anesthesia provider in the mentorship model. The potential mentors were identified and selected by local experts and the Rwandan professional associations. The selected mentors received refresher courses in specific skills in their specialty and a training course on mentorship. Ten teams of five mentors provided support to mentees at their workplace in 10 district hospitals in the Northern and Southern Provinces in Rwanda. By February 2020, 15 visits of 3 consecutive days each had been conducted by the mentoring team in 5 hospitals in the Northern Province from June 2017, while 13 visits were conducted in five hospitals of Southern Province from November 2017. The model was extended to five hospitals in the Southern Province few months later after the introduction of the model in North to ensure the implementation is feasible. A coordination meeting with beneficiaries and implementers identified strategies to overcome the challenges encountered in north. The hospital management team was involved in the development and implementation of the mentorship model to ensure ownership of the model.

### Data collection and analysis

We examined all the reports that were submitted by mentors at the end of the mentoring sessions. Information was extracted and entered into an excel database. Mentors were contacted for further information, corrective measures or clarifications if specific issues were identified from their reports.

### Mentorship model development

To address some of the challenges in further reducing maternal and neonatal morality in Rwanda, the TSAM project team determined that it was essential to implement novel strategies to improve patient outcomes. Thus, a mentorship model was developed to address the limitations of previous approaches to CPD. The model was built on the Rwanda Ministry of Health Guidelines Mentorship [25] with integration of additional components designated as “cross-cutting themes” (CCTs). Initially the CCTs included gender, ethics, and interprofessional collaboration (IPC). As the model was developed two additional themes were included, maternal mental health (MMH) and gender-based violence (GBV). These were added based on consultation with many stakeholders including the University of Rwanda and different professional associations to name a few and current information in Rwanda indicating the high prevalence of post-partum depression and GBV. The different steps followed are provided in Table 1 and the core principles of the TSAM model are provided in Table 2.

**Table 1:** Key steps followed to develop the mentorship model

Number	Key steps
1	Consultations with different stakeholders at different levels
2	Formation of the CPD action team
3	Creation of the post of CPD Manager
4	Development/adaptation of the different tools
5	Selection of potential mentors
6	Refresher skills training of potential mentors
7	Training of mentors on mentoring and Cross Cutting themes
8	Initial field visits in the hospitals
9	Mentorship field visits
10	Different monitoring meetings
11	Additional training of mentors based on the needs

**Table 2:** Core principles of the TSAM mentorship program

Item #	Core Mentorship Principle
1	On-site mentorship by skilled experts
2	Involves interprofessional teams of mentors. Each team included five mentors; a gynecologist/obstetrician, a midwife, a pediatrician, a pediatric nurse and an anesthesiologist.
3	Supported by a platform of ‘cross-cutting themes’ (CCTs).
4	Interdisciplinary team to conduct monthly mentorship visits for three consecutive days. During the lifetime of the project
5	Teams to focus on competency and consistency in mentorship, i.e., mentors would mentor the same mentees at each visit until the mentees reached a level of competency
6	The mentors would receive ongoing support through additional skill development provided through biannual workshops on topics identified by the Action Team.
7	Mentors to support mentees by working side-by-side their mentees.

**Fig. 1:**
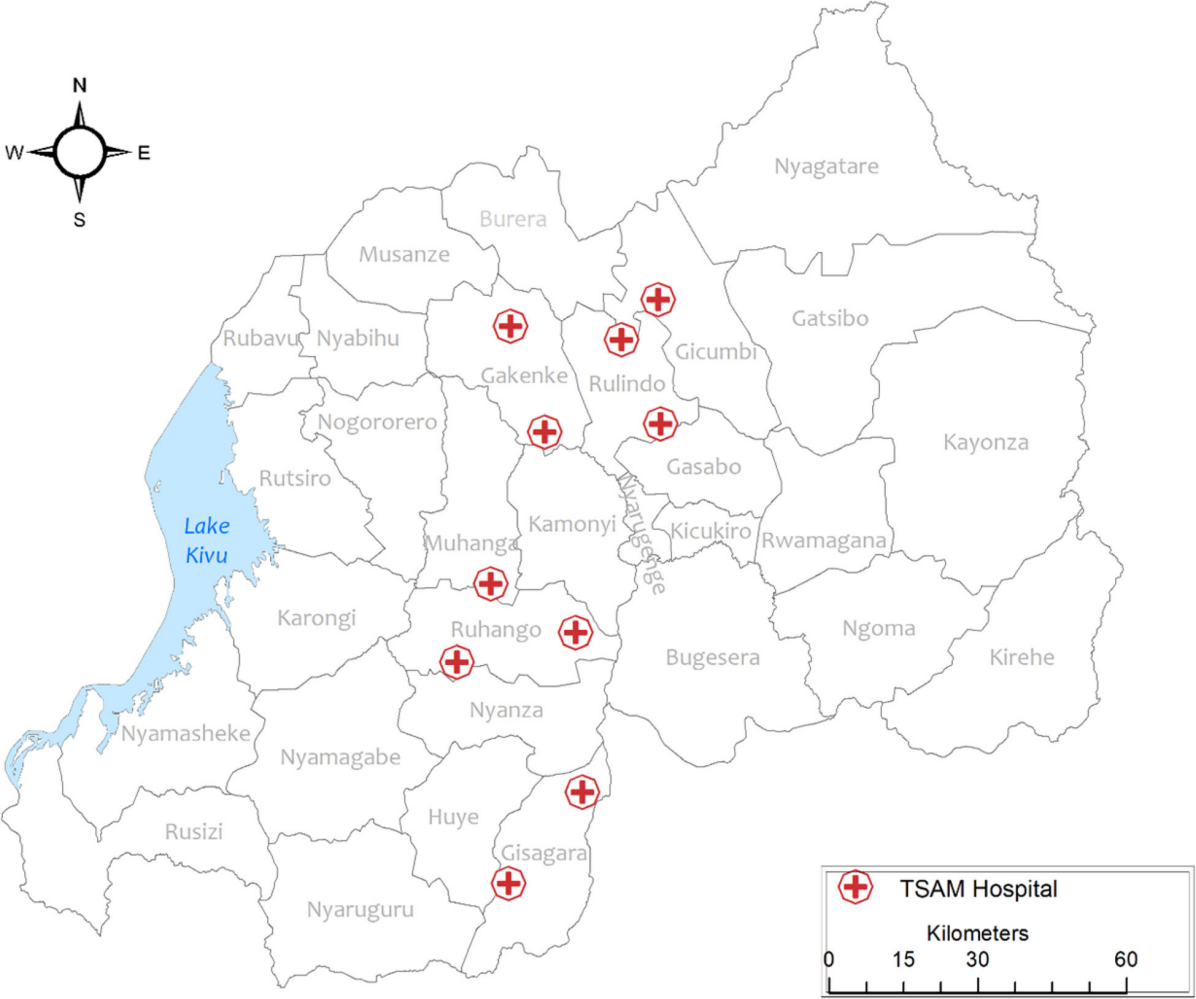
TSAM assigned hospitals in Rwanda. This Figure shows the location of 10 TSAM assigned hospitals where the mentorship model described under this manuscript was implemented. It was produced using ESRI 2019. ArcGIS Desktop: Release 10.7.1. Redlands, CA: Environmental Systems

The region of the country where the mentorship model was to be implemented was determined at the beginning of the project when the Memorandum of Understanding was signed with the Rwanda Ministry of Health (MoH). The MoH assigned TSAM six districts, 3 in the Northern Province (Gakenke, Gicumbi and Rulindo) and 3 in the Southern Province (Muhanga, Ruhango and Gisagara), with five district hospitals in each province (Fig. 1). The model was implemented first in the Northern Province and then refined and taken to the Southern Province.

### Model develeopment

The development of the model involved extended consultation at multiple levels in Rwanda. The model was refined through a series of meetings with officials from the MOH, the provincial governors, the vice mayors who oversee health and social issues, local hospital leadership and administration, and health care providers at the central and local level.

To facilitate collaboration, the management organization included the formation of an “Action Team”. This team brought together representatives of the professional associations; the Director Generals of TSAM assigned hospitals, and some of the senior mentors. To complete the partnership, the Action Team also included project team members from Canada with specific expertise in education and MNCH. The role of the action team was to meet regularly to design and implement the mentorship model, review and adjust the model based on the reports from the mentors, and resolve any issues arising from the implementation.

A position of ‘CPD Manager’ was created to facilitate the development and implementation of the mentorship model and to provide ongoing management and leadership. This was a leadership role that required strong interpersonal skills, a high level of health care knowledge, knowledge of the local context, and experience working with multiple governmental and HCPs ‘institutions. The success of the mentorship program was dependent on the commitment and dedication of the CPD manager to coordinate mentorship activities. The manager’s role included activities such as organizing action team meetings, organizing and mentorship field visits, conducting intermittent field visits to ensure the implementation was smooth, receiving and compiling mentorship reports, sharing the reports to all parties involved to ensure challenges encountered during the visits were identified and strategies to overcome them were developed, organizing additional workshops for mentors and hospital staff and analyzing maternal, newborn and child health data from hospitals to identify trends.

#### Development of the tools

A workshop to review and agree on the tools to be used during the mentorship was conducted in March 2017. This workshop brought together representatives of different professional associations in Rwanda involved in mentoring, managers of TSAM assigned hospitals of the Northern Province and team members from Canada. During this workshop, existing checklists used in mentorship related to MNCH in Rwanda were reviewed and additional checklist items related to the CCT were included. A clinical mentor monthly reporting template was developed. This reporting template included documentation of near misses and critical situations encountered during mentorship visits. Two new forms were created; a form for the mentee to provide feedback to the project on how the mentor was fulfilling his/her tasks and a mentorship reporting form to be used by a mentor describing the progress of mentee.

#### Selection of mentors

Potential national mentors were selected in partnership with the professional associations including Rwanda Society of Obstetricians and Gynecologist which is also a member of FIGO, Rwanda Pediatric Association (RPA), Rwanda Society of Anaesthesiologists (RSA) and Rwanda Associations of Midwives (RAM). This allowed the selection of competent mentors in their area of competency.

Mentors were selected based on the key criteria listed in mentorship guidelines published by the MOH [26]. Using these guidelines, the characteristics of mentors should include (Table 3):

**Table 3:** Characteristics of a Mentor [26]

Number	Characteristic
1	Qualified, competent and experienced in own area of specialization with clinical proficiency and capacity to make decisions
2	Demonstrated willingness to mentor other clinicians through on-site visits
3	Capacity and desire to motivate the mentee to perform well
4	Familiarity with and ability to conduct procedures in accordance with clinical standards and guidelines
5	Ability to facilitate a case discussion
6	Ability to communicate clearly and effectively with staff including provision of constructive, timely, and interactive feedback
7	Ability to gather and analyze data
8	Be a role model and champion of best practices within their own facility
9	Being available and committed to mentorship
10	Demonstrated ability to transfer knowledge and skills
11	Interested in clinical mentorship

#### Refresher skills training for potential mentors

Refresher training was organized for potential mentors. The objective was to provide them with updated clinical skills and to evaluate their suitability as to act as mentors. This two-day refresher training course was conducted in May 2017. The facilitators were specialists representing their professional associations. The facilitators used demonstrations, simulations, discussions, and local protocols to update the skills of the potential mentors. Following the refresher course, the Action Team selected those best suited to become mentors.

#### Training on mentoring and cross cutting themes

In May 2017, a course on mentoring was organized for those selected to become mentors. A team of Canadian medical educational specialists and Rwandan experts facilitated the course. The course covered topics such as the philosophy of mentoring, coaching, teaching a skill, immersive learning models, change theory and changing practice, and giving feedback. The program also introduced the initial three CCTs (IPC, gender and ethics).

In addition to the candidate mentors, the workshop was also attended by the Director Generals, Clinical Directors and Directors of Nursing in TSAM assigned hospitals from the Northern Province. This ensured that the hospital management team understood the mentorship model and would be able to support the mentorship team and their mentees during mentorship visits.

#### Initial field visits of mentors

The initial field visits were conducted the day following the completion of the training on mentoring. Each team of mentors visited their assigned hospital, facilitated by the hospital leadership. The field visits allowed mentors an opportunity to see the facilities and become familiar with the services provided at each hospital. The mentors also conducted a rapid assessment on the availability of services, staff, and key equipment. Another important goal of the initial field visit was ensuring that the staff of the district hospital understood the philosophy and purpose of the mentorship visits.

Prior to the initial field visits, a document was developed and presented to the mentors for guidance while on the field visit. The recommendations in the guiding document included meeting key administrative staff, basic data gathering on the assigned district hospital including availability of staff and equipment, and information about the population served.

### Model implementation

#### Mentorship field visits

After a successful design phase, the first mentorship team visits were organized for the five hospitals in the Northern districts in June 2017 and then in five hospitals of South in November 2017. By the end of February 2020, a total of 15 visits and 13 visits of 3 consecutive days had been conducted by each team. Although there was initial concern about the feasibility of having the five mentors visit as a team this proved not to be problematic. On occasion when one team member could not be available, an experienced mentor replaced that team member for the visit.

As much as possible the model emphasized the one-on-one aspect of mentoring with the expectation that the mentor would work with the same mentee on each visit. This was an early challenge but as the hospital leadership and the mentees grew to appreciate the mentor-mentee relationship this issue was less problematic. During the mentoring period, different key activities were carried out (Table 4).

**Table 4:** Key activities carried out by mentors during the mentorship field activities

Number	Characteristic
1	Discussions on case management with the mentees and other hospital staff
2	Bedside teaching
3	Operating theatre skill reinforcement
4	Presentations on key topics during the morning staff meeting
5	Training of mentees using simulation
6	Participation in deaths surveillance and response
7	Participation in the development and implementation of CQI projects

A key element of the mentoring was that the mentors were not to be considered an extra staff member during the visit but were there to mentor and support their mentees. Upon completion of each visit, the team of mentors provided the feedback to not only mentees but also to the entire hospital staff during the staff meeting on the last day of the visit. The feedback was also provided to the hospital management team with suggestions about what has to be changed and what support is needed by the hospital management team to support the changes. Finally, the team completed their reports, which were then reviewed by the TSAM mentorship manager. The reports were reviewed and analyzed to monitor the mentorship process as well as to maintain a record of activities during mentorship and the feedback was provided to the mentors and hospital management team.

Once the program was functioning in the Northern Province, the experience gained was used to implement the program in the hospitals assigned to TSAM in Southern Province; Gakoma, Gitwe, Kabgayi, Kibirizi and Ruhango.

#### Monitoring and evaluation meetings

To ensure the mentorship approach was running smoothly and experiences were shared, quarterly evaluation meetings were organized. These meetings brought together the Director Generals, Clinical Directors and Directors of Nursing from hospitals benefiting from the mentorship visits as well as TSAM team members involved in the mentorship program. The goal was to share the key messages from the reports of the mentorship visits, discuss the successes and challenges, and to develop strategies to overcome challenges. In addition, mentorship evaluation was always on the agenda of the Action Team meetings. Standardized questionnaires were developed that allowed participants to evaluate all the workshops and courses.

#### Ongoing workshops for mentors

Recognizing that a single workshop on mentoring was not sufficient to build the skills and confidence of mentors, additional workshops were planned. These additional workshops often included the hospital leadership as well. The first one provided in-depth training by Rwandan and Canadian experts on the CCTs.

The second workshop offered was on Maternal Perinatal Death Surveillance and Response (MPDSR) and Continuous Quality Improvement (CQI). The MPDSR is one of the key processes to reduce preventable deaths and an activity to be conducted during mentorship. The workshop was attended by all the mentors and each district hospital was invited to send an additional 3 people from their staff. During the workshop, the mentors and hospital staff worked as a team to develop a potential CQI project that could be done in their hospital. The workshop was facilitated by a CPD team from both Canada and Rwanda, and staff from the Rwanda Biomedical Center, who supervise the death audit process. An additional workshop MPDSR and CQI was organized for the hospital management team recognizing that this team must be supportive of the two processes.

An additional workshop was conducted to expand the CCTs from the original 3 themes of ethics, gender and IPC to include MMH and GBV. The latter 2 themes are an important initiative of the MOH.

The final workshop in the series covered the use of simulation and debriefing to strenghnen the capacity of mentors in transferring the knowledge and skills to their mentees. This workshop reinforced aspects of team functioning and improving team communication and emergency care. The content included development of simulation scenarios and skill stations using mannequins and debriefing after a simulation.

Other topics were suggested by mentors such as leadership training and statistical education to support local evidence-based decision making. It was not possible within the time frame of the project to include all topics. However, the philosophy of providing ongoing skill development for mentors was well received. In addition to regular updates the workshops provided an opportunity for the mentors to get together informally and exchange their experiences.

The final piece of the model development was implementation of a “Train-the Trainer’ approach to ongoing training of new mentors. The mentorship teams enthusiastically embraced this and have conducted workshops on clinical and mentorship skills for new mentors to expand the team of available mentors.

## Results

### Health care providers trained

The mentorship visits mentioned in the Methods, resulted in a total of two hundred and eighteen (218) HCPs who benefited from the program as mentees. To be described as a mentee, a health care provider should have attended at least two visits of 3 consecutive days each. The mentees were comprised of a mix of HPCs including 81 physicians (37.1%), 60 midwives (27.5%), 47 nurses (21.6%) and 30 non-physician anaesthesia providers (13.8%) all involved in proving MNCH care in district hospitals. The health care providers practiced in different services including maternity, pediatrics, neonatology, and the operating room. With regards to gender, among 218 mentees, 115 (52.8%) were females while 103 were males (47.2%). The details on the characteristics of mentees are shown in Table 5.

**Table 5:** Characteristics of mentees by the end 13 visits in South and 15 visits in North

Profession	Physicians		Midwives		Nurses		NPA		Total
Sex/Province	N	S	N	S	N	S	N	S	N + S
Males	38	31	7	2	3	2	14	6	103 (47.2%)
Female	9	3	27	24	26	16	4	6	115 (52.8%)
Sub total	47	34	34	26	29	18	18	12	
Grand total	81 (37.1%)		60 (27.5%)		47 (21.6%)		30 (13.8%)		218 (100%)

*N* North, *S* South

### Cases managed by mentors and mentees

Reports generated to date, highlighted the fact that during the mentorship visits, the lives of several mothers and newborns were saved when they experienced a ‘near miss’ event. These cases were considered excellent examples of how a team of HCPs is effective in managing difficult emergency cases and this suggested an early positive impact of the model. These cases gave the local health care providers and the mentorship team an opportunity to experience the satisfaction of working together as a team to achieve a positive life-saving outcome.

“We saved the mother in hypovolemic shock after delivery because the composition of the team was multidisciplinary with the availability of experienced anesthesiologist which helped to stabilized the mother first and then the gynecologist obstetrician who conducted the hysterectomy in the presence of experienced anesthesia provider” a team of mentors from different hospitals said”.

### Presentations conducted

For all mentorship visits, the mentors prepared presentations on the key topics focusing on the diagnosis and management of most emergency maternal, newborn and child health conditions. These presentations benefited many staff members of the hospitals as they were given to morning staff meetings allowing the staff to increase their knowledge and skills in management of most fatal maternal, newborn and child incidences.

### Lessons learned from the mentorship model and key challenges

Throughout the process of providing the training and delivering the mentoring, the project learned several valuable lessons related to the effectiveness of the model. These included:

- The mentorship model built on IPC is very helpful and appreciated by the beneficiaries of the mentoring at the district hospitals
- Many cases that qualified as near misses were saved during the mentoring field visits and in most of the cases, the patient was saved as a result of the IPC team composition
- The involvement of the hospital management team is crucial for mentorship at all levels to achieve its goals.
- The non-physicians anesthetists working at the DH level need to participate in the mentorship program to ensure their capacities are strengthened to ensure safe obstetric practice

Apart from the potential benefit and positive lessons learned, some challenges were faced and should be overcome for a better integration of the model in the existing health system structure. These challenges included the limited number of HCPs in hospitals which impede training big number of mentees at the same time. The model encouraged continuous peer mentoring outside the normal mentorship visits to ensure the knowledge and skills gained by mentees are transferred to their workmates. The other challenge was the high staff turnover of health care providers across health facilities which lead to the inconsistency of mentees during the mentorship visits.

### Ongoing research and implication on future research

This mentorship model development resulted in several important findings that will be published in future focusing on the perceptions of mentors/mentors on the mentorship model, the role of interprofessional collaboration in designing and implementing the mentorship model, and the impact of the model. With regards to future studies, it would be important to examine the relationship between the effects of mentorship on job retention of mentees given that mentees seemed to be rather mobile following mentorship training, and given that job satisfaction and job retention among HCPs as career development is one of the determinants of job satisfaction and job retention [11]. The cost effectiveness of the onsite mentorship should also be examined.

## Discussion

To our knowledge this is the first demonstration of the implementation of this type of a mentorship model in the region The model utilizes several unique features including a multi-disciplinary team, CCTs such as ethics, gender, IPC, MMH and GBV, and regular 3-day visits to each district hospital.

The implementation of this model has been enthusiastically accepted by the mentors, the mentees and hospital management teams. The positive early results at the district hospital level of a mentorship model built on a foundation of IPC suggest this approach has been successful. Health care providers appreciated the composition of a mentorship team that included different professions. This demonstrated to the district hospital staff that working as a team can result in better outcomes for mothers and newborns.

The early constraints included the inconsistency of mentees. This issue was discussed during the monitoring meetings. This progressively improved as mentees realized the mentorship program was helping them to improve their competency. They appreciated that mentorship was being conducted in a collaborative manner, rather than the classical teaching and punitive supervision they may have experienced in the past. However, staff turnover continues to complicate the program, and other strategies for retention in addition to the mentorship program are required.

The nature of the ‘near-miss’ cases encountered during the mentorship visits illustrated the importance of an experienced interdisciplinary team to stabilize these critically ill patients. We felt this reinforced the need to support a model of mentorship built on IPC with a strong emphasis on professional ethics and gender equality.

Nevertheless, the composition of the team is not itself sufficient. The team members must be trained on key concepts which include IPC for the approach to be most successful. In addition, ownership by the health facility team is critical to support mentorship. The TSAM mentorship program developed ownership by the health facility by inviting the administration of the hospital to several different workshops and meetings, involved local leadership in decision-making, invited their feedback, and maintained regular contact with them.

### Strengths and limitations of your study

The strengths of this study include the development of a national mentorship model that has been adopted by the professional associations in Rwanda and have been used in the selection of the competent and committed mentors in their areas of competency. Furthermore, the commitment of the leadership of the various hospitals was also crucial in the attainment of the mentorship objectives. Strength of this model was the collaborative involvement of our Canadian colleagues, which allowed them to share their expertise in several areas on maternal and newborn health with the local teams. Also, the number of mentorship visits has been enough to build the capacities of HCPs.

Despite the impact of this mentorship model, there were a number of limitations worth noting. These included the fact that mentors were not only engaged full-time in the mentoring program and sometimes their others schedules interfered their mentorship field visits. In such cases the TSAM team tried to call on reserve mentors but this then affected the smooth running and continuity of the program. This also hindered the relationships that might have been built between mentors and mentees, which is the cornerstone of the mentoring program. Based on this, we recommend that mentorship programs in similar context should always strive to recruit mentors for specified time when they can fully dedicate their time for mentoring. Similarly, with regards to the mentees, a key limitation was that they were usually too busy with their routine activities which frequently interfered with the learning activities by the mentoring program. This had negative impacts on some of the mentees related to the completion of the program that the mentor had planned to cover during the mentoring field visit. In this regard, we recommend that selected mentees should be released from their normal schedules during field visits of mentors to be able to concentrate on the program activities. Another challenge was the frequent turnover of some mentees. When mentees left in the middle of the programs, the mentors had to start the process all over again with new mentees, and this also disrupted the continuity of the programme and the development of competencies. We recommend that hospital administrators would need to work with mentees on issues related to retention to avoid mentees leaving after they have acquired needed knowledge for the provision of quality maternal, newborn and child health care. Finally, mentors benefited the allowances and it may be difficult to sustain the program. The recommendation would be to integrate the mentorship activities in the routine activities of the referral hospitals.

## Conclusion

The initiation of a mentorship model built on IPC and focused on HCPs involved in maternal and perinatal care can lead to valuable training of a considerable number of HCPs. Effective leadership at the district hospital is key to successful implementation. A dedicated program leader-manager is essential to implement such a program and to maintain success.

In conclusion, in LIC settings where maternal and perinatal deaths are high and there is a lack of specialized health care providers, a mentorship model with mentors from a mix of health professionals, together with strong health care leadership can yield positive results.

## Data Availability

The datasets and other tools used in this study may be available from the corresponding author upon a reasonable request.

## Abbreviations

CCTs: Cross Cutting themes
CPD: Continous Professional Development
DH: District Hospital
EmONC: Courses on emergency, obstetric neonatal care
GAC: Global Affairs Canada
GBV: Gender Based Violence
HCP: Health care providers
IPC: Inter-Professional Collaboration
LIC: Low Income Country
MDGs: Millennium Development Goals
MMR: Maternal Mortality Ratio
MNC: Maternal Newborn Care
MNCH: Maternal Newborn Child Health
MoU: Memorandum of Understanding
NPA: Non-Physician Anesthetist
PI: Principal Investigator
TSAM: Training Support and Access Model
WHO: World Health Organization
